# Validity of the Brief Resilience Scale and Brief Resilient Coping Scale in a Chinese Sample

**DOI:** 10.3390/ijerph17041265

**Published:** 2020-02-16

**Authors:** Sai-fu Fung

**Affiliations:** Department of Social and Behavioural Sciences, City University of Hong Kong, Hong Kong, China; sffung@cityu.edu.hk

**Keywords:** BRS, BRCS, Chinese, resilience, university student, confirmatory factor analysis

## Abstract

This study presents a cross-cultural examination of the psychometric properties of two commonly used brief self-report resilience scales, the 6-item Brief Resilience Scale (BRS) and the 4-item Brief Resilient Coping Scale (BRCS). Five hundred and eleven Chinese university undergraduate students were recruited for this cross-sectional research. Various psychometric evaluation tools were used to evaluate the internal consistency, criterion validity, factorial validity and construct validity of these resilience scales. The results showed that both scales had good criterion validity, with well-established measures of well-being, optimism, self-esteem, self-efficacy and mental health, as suggested in the resilience literature. The BRS (*a* = 0.71) showed better internal consistency than the BRCS (*a* = 0.59). The confirmatory factor analysis (CFA) results also indicated that the BRS, with a two-factor structure, had better construct validity than the BRCS. The CFA results for the BRS met all of the criteria for a good model fit. The BRS was found to have better psychometric properties than the BRCS in the Chinese context. The findings will help researchers to select an appropriate resilience measure when conducting epistemological surveys of Chinese university students or the Chinese diaspora in other contexts.

## 1. Introduction

“Resilience” refers to “the ability to bounce back” [1], which is an important concept in the field of social and behavioural sciences. In recent decades, resilience has been widely discussed and examined by identifying the individual protective factors that foster positive adaptation, coping styles, cognitive problem-solving skills, reflective thinking skills to manage problems and the ability to stimulate positive support from others [1,2,3,4,5,6]. A resilient individual is characterised as having a positive view of stress and the ability to manage stress effectively, adapt to change and cope with adverse conditions, including catastrophic life events, socioeconomic disadvantage and mental and chronic illness [7,8,9]. Recent studies have shown that resilience has a particularly positive effect on the quality-of-life of people with terminal illness or post-traumatic stress [10,11].

Resilience is also considered a measure of stress-coping ability, and as such can be used by clinical practitioners to evaluate treatment for anxiety and depression [12]. Among the commonly used resilience measures, the most popular, the Brief Resilience Scale (BRS) [1], derived from the work of Carver [4], and the Brief Resilient Coping Scale (BRCS) [3], theoretically based on Polk [6], are concise, with only six and four items, respectively [13,14]. Other renowned resilience scales are much longer, ranging from the 10-item Connor–Davidson Resilience Scale [15] to the 102-item Ego Resiliency Scale [16]. However, the application of these lengthy scales may be limited because of the time-consuming data collection process, which “may result in high rates of non-response of missing data” [14]. Hence, this study compared the psychometric properties of two relatively short resilience measures, namely the BRS and the BRCS.

Both scales have been translated into different languages and adapted for different study samples. In recent years, BRS validation studies have been carried out in Brazil on middle-class young adults (α = 0.76) [17], the general adult population in Germany (α = 0.85) [18,19], Spanish adults with various medical conditions (α = 0.83) [20], blue- and white-collar workers in the Netherlands (α = 0.78) [21] and Mexican and Chilean university students (α = 0.71 to 0.77) [22]. The BRS has been used in research populations such as cancer patients [23] and vocational rehabilitation service recipients [24]. Similarly, BRCS validation studies have been performed in different languages and with different groups, such as Spanish university students (α = 0.67) [25] and a household survey sample in Germany (α = 0.78) [26]. In addition, the BRCS has been used to study patients with systemic lupus erythematosus [27].

This study contributes to research in the following ways. Despite the popularity of these two resilience scales, few studies have validated the BRS and the BRCS in China. The literature on resilience scales has compared these scales using secondary data only [13,14]. Therefore, this pioneering study aimed to validate and compare the psychometric properties of these two brief resilience measures using empirical data to provide a point of reference for practitioners and researchers in the field.

## 2. Materials and Methods

### 2.1. Participants

Five hundred and eleven undergraduate students from a university in Guangzhou, China, were recruited to take part in this cross-sectional study. The respondents were 20.41 years old on average (SD = 2.49). The sample included 14.5% male and 85.5% female respondents, which reflected the general demographic profile of this university. Data collection took place in April and May 2019 via the university’s intranet system using an innovative self-report smartphone-based application. The students were invited to participate in the study on a voluntary basis. All respondents gave their informed consent for inclusion before participating in the study. The study was conducted in accordance with the Declaration of Helsinki and the regulations stipulated in Article 14 of Chapter III, Statistics Law of the People’s Republic of China, and the protocol was approved by the Ethics Committee of Huashang College, Guangdong University of Business Studies.

### 2.2. Measures

The BRS includes six items. The respondents were asked to indicate how well each statement described their behaviour and actions on a 5-point Likert-type scale, ranging from “1” = *does not describe me at all* to “5” = *describes me very well*. As Item 2 (*I have a hard time making it through stressful events*), Item 4 (*It is hard for me to snap back when something bad happens*) and Item 6 (*I tend to take a long time to get over set-backs in my life*) were reverse-coded, the data collected were recoded prior to analysis [1]. The BRCS has four items. The participants were asked to select one option for each statement to indicate their level of disagreement or agreement with each item on a 5-point Likert-type scale, ranging from 1 = *strongly disagree* to 5 = *strongly agree* [3]. The items from each scale were translated into Chinese and verified using the back-translation procedure by two translators fluent in both Chinese and English. Two pilot studies were conducted in the Shaanxi (*n* = 5) and Guangdong (*n* = 5) provinces to ensure that the translated versions were free of any geographic and cultural differences in China [28,29,30]. None of the participants in the pilot study reported difficulties understanding and answering the questionnaires. The data from the pilot studies were not included in this study.

### 2.3. Procedure

Various psychometric analyses were used in this study. The internal consistency of each scale was assessed using Cronbach’s alpha [31] and by examining the corrected item-total correlations between the individual items [32,33]. Criterion-related validity was evaluated using other resilience-related constructs reported in the literature. Recent studies have shown that resilience measures are positively linked to mental health measures, optimism, coping strategies, self-esteem, self-efficacy, well-being and life satisfaction [17,18,19,25]. Therefore, this study used several well-established scales to evaluate the criterion validity of the BRS and the BRCS, including the Satisfaction with Life Scale (SWLS) [34,35,36,37], the WHO (Five) Well-being Index (WHO-5) [38,39,40], the Revised Life Orientation Test (LOT-R) [41,42,43], the General Self-efficacy Scale (GSE) [44,45,46], the Rosenberg Self-esteem (RSE) Scale [47,48], the Short Warwick Edinburgh Mental Well-being Scale (SWEMWBS) [49,50,51] and positive affect in the Positive and Negative Affect Schedule (PANAS) [52,53,54]. However, the scales were expected to be negatively associated with anxiety, depression and negative coping strategies [19,20,25]. Hence, we used the 12-item General Health Questionnaire (GHQ-12) [55,56,57] and the negative affect in the PANAS [52,53,54] to evaluate the relationship between eudaimonic well-being, health-related symptoms and negative affect with the two resilience scales.

The factor structures of the BRS and the BRCS were evaluated using the Kaiser–Meyer–Olkin (KMO) measure and Bartlett’s test of sphericity. The KMO estimates were above 0.70 and the results of Bartlett’s test were significant (*p* < 0.01), indicating that the scales had satisfactory factor structures [58]. Confirmatory factor analysis (CFA) was used to evaluate the construct validity of the scales [59,60,61]. Because the BRS and BRCS include ordinal items, the diagonally weighted least squares CFA estimator was used to construct the latent factor structures [62,63,64]. The following cut-off criteria were adopted from the structural equation modelling literature to indicate an adequate model fit: CFI > 0.95, TLI > 0.95, RMSEA < 0.06, SRMR < 0.08 [32,59,65,66]. In addition to these measures, a chi square/df ratio less than 3 was used to determine the model fit [67,68,69,70]. The analyses were computed using IBM SPSS 25.0 and R version 3.6.0 with the lavaan package 0.6-3 [71].

## 3. Results

### 3.1. Internal Consistency

Table 1 presents the means, standard deviations, skewness values, kurtosis values, corrected item-total correlations and alpha if the item was deleted, for the 6-item BRS and the 4-item BRCS (*N* = 511). The total mean score for the BRS and the BRCS was 19.85 (SD = 3.31) and 13.29 (SD = 2.19), respectively. The corrected item-total correlations for individual items in both scales were above 0.30, indicating their suitability for scale construction [32,33]. Those for the BRS ranged from 0.33 to 0.53, while those for the BRCS ranged from 0.31 to 0.42 (Table 1). However, only the BRS was found to have an acceptable Cronbach’s alpha value (0.71), while the BRCS yielded a problematic value of coefficient alpha of 0.59.

### 3.2. Convergent Validity

The results showed that both resilience scales replicated the correlational direction and magnitude of other well-established measures of well-being, optimism, self-esteem, self-efficacy and mental health. According to Table 2, significant and moderate positive correlations were observed between the BRS and the SWLS (*r* = 0.23, *p* < 0.001), the WHO-5 (*r* = 0.30, *p* < 0.001), the LOT-R (*r* = 0.30, *p* < 0.001), the RSE (*r* = 0.44, *p* < 0.001), the GSE (*r* = 0.29, *p* < 0.001), the SWEMWBS (*r* = 0.45, *p* < 0.001) and positive affect in the PANAS (*r* = 0.26, *p* < 0.001). The BRCS had significant and moderate positive correlations with the SWLS (*r* = 0.35, *p* < 0.001), the WHO-5 (*r* = 0.29, *p* < 0.001), the LOT-R (*r* = 0.39, *p* < 0.001), the RSE (*r* = 0.34, *p* < 0.001), the GSE (*r* = 0.42, *p* < 0.001), the SWEMWBS (*r* = 0.44, *p* < 0.001) and positive affect in the PANAS (*r* = 0.40, *p* < 0.001).

The BRCS had a significantly weak to moderate negative correlations with the GHQ-12 (*r* = −0.33, *p* < 0.001) and negative affect in the PANAS (*r* = −0.13, *p* = 0.003), while the BRS had a significant and moderate positive relationship with the GHQ-12 (*r* = −0.50, *p* < 0.001) and negative affect in the PANAS (*r* = −0.41, *p* < 0.001).

### 3.3. Factorial Structure

**Figure 1:**
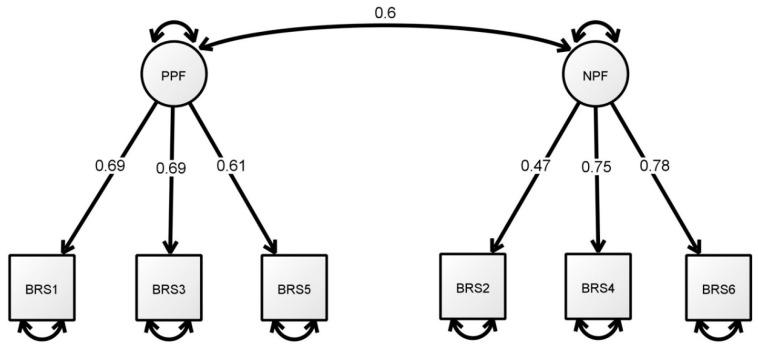
The final standardised model of the two-factor BRS. Note. PPF = positive polarity factor; NPF = negative polarity factor.

The factor analysis results yielded KMO values of 0.75 for the BRS (χ^2^ = 537.114, *p* < 0.001) and 0.65 (χ^2^ = 198.411, *p* < 0.001) for the BRCS. Table 3 shows the CFA results for the 6-item BRS and the 4-item BRCS. The results for Model 1, the CFA of the BRS with uncorrelated error terms, indicated a poor model fit: χ^2^ (120.680)/9 = 13.41, RMSEA = 0.156, SRMR = 0.082 and TLI = 0.893. Model 2 re-evaluated the unidimensional BRS with correlated errors based on the modification indices. The results indicated a good model fit, with χ^2^ (11.787)/6 = 1.96, *p* = 0.067, SRMR = 0.028, CFI = 0.997, TLI = 0.992 and RMSEA = 0.043. Model 3 tested the 6-item BRS with a two-factor structure, including the positive polarity factor (PPF; BRS1, BRS3 and BRS5) and the negative polarity factor (NPF; BRS2, BRS4 and BRS6) without correlating the error terms [21,22]. The results showed that Model 3 reached all cut-off values for a good fit, with χ^2^ (13.681)/8 = 1.71, *p* = 0.090, SRMR = 0.030, CFI = 0.997, TLI = 0.994 and RMSEA = 0.037 (Figure 1).

Model 4 evaluated the BRCS without correlating the measurement errors. The results of Model 4 revealed a poor model fit, with χ^2^ (17.538)/2 = 8.77, TLI = 0.896 and RMSEA = 0.123. Based on the modification indices, Model 5 included covariance factors between the error terms for items BRCS1 and BRCS2. The results indicated a good model fit, with χ^2^ (0.81)/1 = 0.807, *p* = 0.369, SRMR = 0.011, CFI = 0.999, TLI = 0.999 and RMSEA < 0.001. Overall, these results showed that the 6-item BRS with a two-factor structure had a better factorial and construct validity than the unidimensional BRS and the 4-item BRCS.

## 4. Discussion

The BRS in this study generally showed better psychometric properties than the BRCS. The BRS had a good internal consistency, with a coefficient alpha of 0.71. This result is consistent with the alpha values (ranging from 0.71 to 0.85) reported in other BRS validation studies of workers, university students and patients with cancer or heart conditions in Germany, the Netherlands, Spain and the United States [1,18,19,20,21,22]. In contrast, the results for the BRCS indicated a poor Cronbach’s alpha value (0.59). This result was expected as the original scale, which was validated in a longitudinal study with 230 respondents from two samples, also had a marginal coefficient alpha, ranging from 0.64 to 0.76 [3]. The results also suggested that the BRS had a better factor structure than the BRCS. The CFA results indicated that only the 6-item BRS, with a two-factor structure (Model 3), met all of the criteria for a good model fit without correlating the error terms between the items.

The two-factor structure of the BRS has been well supported and discussed in the literature. The original BRS was proposed to be unidimensional with negative items (2, 4 and 6). The scale was developed solely based on the results of exploratory factor analysis (EFA) from four samples [1]. However, many subsequent BRS studies have used CFA to verify the underlying factor structure of the scale, suggesting that it comprises two latent factors, namely the positive items (1, 3 and 5) related to resilience and the negative valence items (2, 4 and 6) related to succumbing [17,20,24]. This phenomenon has been highlighted and justified in the BRS literature based on the effects of the use of reversed items on workers or samples from developing countries [21,22].

This study has several limitations. The sampling method and sample size may limit the reliability of the results. The sample consisted of 511 undergraduate students from a single Chinese university in Guangzhou. Most of the students majored in accountancy, business, computer science or arts and humanities. The concept of resilience has been used primarily in studies of patients with medical conditions. However, this study was unable to recruit a sample from a clinical setting, which may limit the extrapolation of the results to other contexts. Nevertheless, the original BRS was developed based on only 195 American undergraduate students [1]. As a result, the sampling method and sample size for this study were the same as those in the original study. The results of this study also replicated the results of previous BRS studies (e.g., the structure with two latent factors and Cronbach’s alpha ranging from 0.71 to 0.85), including those with samples from clinical settings [17,18,19,20,21,22,24].

Another limitation is related to the use of resilience-related constructs when evaluating the criterion validity of the resilience scales. The BRS and BRCS literature has adopted various scales to analyse the criterion validity of these scales, namely the Connor–Davidson Resilience Scale [1,20], the Brief COP [1,19], the Hospital Anxiety and Depression Scale [1,20] and the Optimism/Pessimism Scale [18,19]. To avoid using a long questionnaire in a validated Chinese version, this study used the following renowned resilience-related constructs, also used in the BRS and BRCS literature: the SWLS [3,24], the WHO-5 [19], the LOT-R [1,3], the GSE [3,18], the PANAS [1,3] and the GHQ-12 [19,21]. The results show that the BRS and the BRCS have good criterion validity.

## 5. Conclusions

This cross-cultural validation study compared the psychometric properties of the BRS and the BRCS in Chinese university students. These scales are the most concise of the commonly used scales to measure resilience. The results suggest that both scales have good criterion validity, with well-established measures of well-being, optimism, self-esteem, self-efficacy and mental health, as suggested in the resilience literature. The factor structure and psychometric properties of the 6-item BRS are better than those of the 4-item BRCS. Therefore, researchers in clinical practice should find the BRS a handy tool for use in epistemological surveys and for evaluating the effectiveness of intervention programmes.

## Figures and Tables

**Table 1:** Descriptive statistics of the Brief Resilience Scale (BRS) and Brief Resilient Coping Scale (BRCS) items.

Item	M	SD	sk	ku	r*_it_*	*a_iid_*
BRS (a *=* 0.71)						
1. I tend to bounce back quickly after hard times	3.21	0.87	−0.34	0.30	0.44	0.67
2. I have a hard time making it through stressful events (R)	3.34	0.93	−0.35	−0.09	0.33	0.71
3. It does not take me long to recover from a stressful event	3.15	0.85	−0.16	0.09	0.44	0.67
4. It is hard for me to snap back when something bad happens (R)	3.38	0.91	−0.45	0.06	0.50	0.65
5. I usually come through difficult times with little trouble	3.51	0.76	−0.27	0.39	0.41	0.68
6. I tend to take a long time to get over set−backs in my life (R)	3.25	0.86	−0.37	0.18	0.53	0.64
Total score	19.85	3.31	−0.52	0.58		
BRCS (a = 0.59)						
1. I look for creative ways to alter difficult situations	3.10	0.79	−0.27	0.49	0.36	0.53
2. Regardless of what happens to me, I believe I can control my reaction to it	3.27	0.83	−0.08	0.16	0.40	0.50
3. I believe that I can grow in positive ways by dealing with difficult situations	3.59	0.82	−0.47	0.43	0.42	0.48
4. I actively look for ways to replace the losses I encounter in life	3.33	0.83	−0.21	0.18	0.31	0.57
Total score	13.29	2.19	−0.98	0.59		

Note. (R) = Reversed item; sk = Skewness; ku = Kurtosis; r*_it_* = Corrected item-total correlations; *a_iid_* = Cronbach’s alpha, if the item is deleted.

**Table 2:** Pearson correlations between the BRS and BRCS compared with other well-established scales.

Scale	BRS *r*	BRCS *r*
Satisfaction with Life Scale (SWLS)	0.23 ***	0.35 ***
WHO (Five) Well-being Index (WHO-5)	0.30 ***	0.29 ***
Revised Life Orientation Test (LOT-R)	0.30 ***	0.39 ***
Rosenberg Self-esteem (RSE) Scale	0.44 ***	0.34 ***
General Self-efficacy Scale (GSE)	0.29 ***	0.42 ***
Short Warwick Edinburgh Mental Well-being Scale (SWEMWBS)	0.45 ***	0.44 ***
Positive affect–PANAS	0.26 ***	0.40 ***
12-item General Health Questionnaire (GHQ-12)	−0.50 ***	−0.33 ***
Negative affect–PANAS	−0.41 ***	−0.13 **

Note. ** *p* < 0.01. *** *p* < 0.001.

**Table 3:** Confirmatory Factor Analysis of the BRS and the BRCS.

Model	χ^2^	df	χ^2^/df	RMSEA (90% CI)	CFI	TLI	SRMR
BRS							
1	120.680	9	13.41	0.156 (0.132–0.181)	0.936	0.893	0.082
2 ^a^	11.787	6	1.96	0.043 (0.000–0.080)	0.997	0.992	0.028
3	13.681	8	1.71	0.037 (0.000–0.070)	0.997	0.994	0.030
BRCS							
4	17.538	2	8.77	0.123 (0.075–0.179)	0.965	0.896	0.050
5 ^b^	0.807	1	0.81	0.000 (0.000–0.112)	0.999	0.999	0.011
Acceptable cut-off value			<3	<0.06	>0.95	>0.95	<0.08

Note. ^a^ Includes the covariance between the error terms for items BRS1 and BRS3, BRS3 and BRS5, BRS1 and BRS5. ^b^ Includes the covariance between the error terms for items BRCS1and BRCS2.
